# Competition between diagonal and off-diagonal coupling gives rise to charge-transfer states in polymeric solar cells

**DOI:** 10.1038/srep14555

**Published:** 2015-09-28

**Authors:** Yao Yao, Nengji Zhou, Javier Prior, Yang Zhao

**Affiliations:** 1 State Key Laboratory of Surface Physics and Department of Physics, Fudan University, Shanghai 200433, China; 2 Collaborative Innovation Center of Advanced Microstructures, Fudan University, Shanghai 200433, China; 3 Division of Materials Science, Nanyang Technological University, Singapore 639798, Singapore; 4 Department of Physics, Hangzhou Normal University, Hangzhou 310036, China; 5 Departamento de Física Aplicada, Universidad Politécnica de Cartagena, Cartagena 30202, Spain

## Abstract

It has long been a puzzle on what drives charge separation in artificial polymeric solar cells as a consensus has yet to emerge among rivaling theories based upon electronic localization and delocalization pictures. Here we propose an alternative using the two-bath spin-boson model with simultaneous diagonal and off-diagonal coupling: the critical phase, which is born out of the competition of the two coupling types, and is neither localized nor delocalized. The decoherence-free feature of the critical phase also helps explain sustained coherence of the charge-transfer state. Exploiting Hamiltonian symmetries in an enhanced algorithm of density-matrix renormalization group, we map out boundaries of the critical phase to a precision previously unattainable, and determine the bath spectral densities inducive to the existence of the charge-transfer state.

The power conversion efficiency (PCE) of polymeric solar cells (PSCs), currently exceeding 10%, has reached the threshold for industrial applications^1^. Closer attention is now being paid to explore the intrinsic mechanisms responsible for increased PCE. Center to the complicated process of charge photogeneration is the charge separation of excitons^2^ and the driving force behind it, attracting great interest from experimentalists^3^,^4^,^5^,^6^,^7^,^8^ and theorists^9^,^10^,^11^ alike. Contention surrounds two candidates that have been proposed and partially corroborated by measurements: the pump-push-probe experiment highlighted the delocalized charge-transfer (CT) state as the essential intermediary between the exciton and the charge-separated (CS) state^3^, while the entropic considerations lead to localized molecular energy levels and the incoherent hopping mechanism with an emphasis on the three-dimensional structure of the acceptors^4^ and a larger entropy for the CS state (than the excitonic state)^2^,^11^. Seemingly incompatible with each other, the delocalization and localization pictures of charge separation await resolutions by future experiments.

Recently, an ultrafast mode of quantum beating with a period of ~23 fs was revealed in a typical PSC structure^8^. As the oscillation timescale is close to those of the stretch mode of polymers and the pinch mode of fullerene, it is conjectured that the vibrational modes instead of the transfer integral of electronic orbitals matters in the oscillations^12^, a conclusion not unseen in photosynthetic light-harvesting systems^13^,^14^,^15^. Organic materials are known to have moderate to strong electron-phonon interactions^16^. As predicted by polaron theory, intramolecular modes induce localization^17^, while dynamic disorder, contributed by the intermolecular modes, leads to simultaneous localization and delocalization^18^,^19^,^20^. The CT state in organic materials is of particular interest thanks to its coupling to both intramolecular (diagonal) and intermolecular (off-diagonal) phonons^17^. Superior diagonal coupling gives rise to the geminate recombination from the CT state to the exciton, while dominating off-diagonal coupling converts the CT state to the CS state^2^. From this perspective, in either scenario, the CT state seems to be an short-lived transient state in the charge separation process. Red-shifted photoluminescence measurements have demonstrated a long-lived CT state in most commonly-used polymers^21^,^22^,^23^. It then implies that, neither localized nor delocalized, the CT state is a “critical” state in between, which is a relatively stable state of the vibronically-coupled system.

In the literature, an excitonic paradigm often contains a two-level system coupled to its environment made of boson modes^24^,^25^. Borrowing the language of open systems, this leads to the celebrated spin-boson model (SBM)^26^,^27^, a convenient venue to study the phase transition between the localized and delocalized phases, or from a slightly different perspective, the dynamical phase transition between the coherent and incoherent phases^28^,^29^,^30^,^31^,^32^,^33^,^34^,^35^,^36^,^37^. As corroborated by the experiment of the singlet fission of excitons^38^, both the intra- and inter-molecular vibronic couplings are equally important^39^,^40^. More interesting to consider is thus the Munn-Silbey approach to simultaneous diagonal and off-diagonal coupling in molecular crystals^18^,^19^,^41^, which states that the diffusion coefficient of charge carriers in molecular crystals is dominated by the competition between the two forms of vibronic coupling. Inspired by recent discovery on the novelty of the CT state^8^, we report in this paper our findings on the two-bath SBM (TBSBM) after taking into account^42^ simultaneous diagonal and off-diagonal coupling. In particular, we focus on results from a critical phase born out of the competition between diagonal and off-diagonal coupling, therefore setting up a benchmarking microscopic model for the study of the charge separation process in PSCs.

## Results

### Two-bath spin-boson model

Despite its simplicity, the SBM is a highly nontrivial model in nearly all aspects, and currently contention still surrounds the existence and the precise locations of phase transitions. Designed to help understand intrinsic mechanisms of coherent exciton dynamics, every theoretical approach typically works accurately only in a certain applicable regime, which is often far away from the critical point, preventing the method from addressing issues related to the phase transition. In this paper, we consider the TBSBM in which a two-level system (a spin) is coupled diagonally and off-diagonally to two independent vibrational baths characterized by continuum spectral densities^42^. The corresponding Hamiltonian can be written as


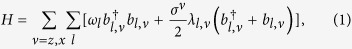


where *σ*^*z*^ and *σ*^*x*^ are the Pauli operators, 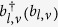 is the creation (annihilation) operator of the *l*-th mode of frequency *ω*_*l*_ in the *ν*-th bath (*ν* = *z*, *x*), and *λ*_*l*,*ν*_ represents the corresponding spin-bath coupling strength. In the traditional SBM, the bath spectral density has a cut-off frequency *ω*_*c*_. For simplicity, the same cut-off frequency is assigned to the spectral density functions of the two baths in this work, i.e., 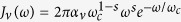 with *α*_*ν*_ being the dimensionless coupling strength for the *ν* bath and *s* being the exponent. We will focus on the case of *s* < 1 corresponding to the sub-Ohmic regime which gives higher weights to low-frequency vibrational modes, as featured in organic materials. It is noted that the behavior of the model could be significantly different if other forms of spectral density were found to be appropriate.

**Figure 1:**
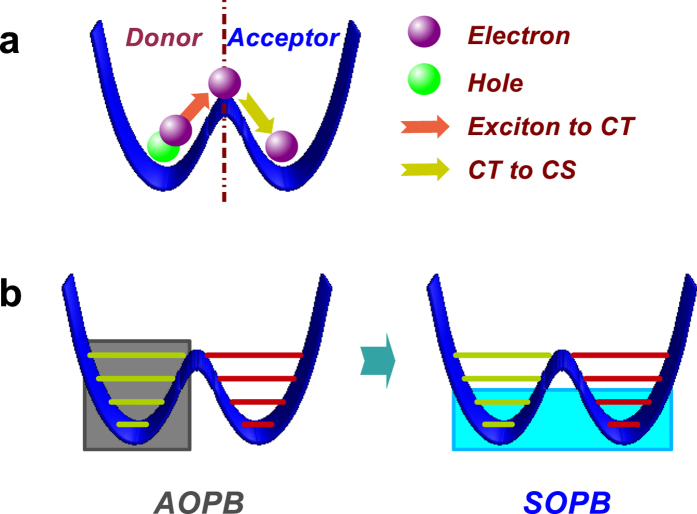
Schematics. (**a**) Schematic for the correspondence between the phases of TBSBM and the states in charge separation. In the localized phase, the spin is polarized in one potential valley formed by the vibrational modes, corresponding to the situation that the electron and hole are strongly bound with each other. In the delocalized phase, the spin is equally populated in two valleys, equivalent to the case that the coherence of electron and hole is completely quenched. In between, both the local and nonlocal coherence are retained. (**b**) The two kinds of optimal phonon basis for the DMRG calculations. The AOPB is shown which takes into account the phonon bases with shifted displacements^43^. The SOPB is shown which considers the globally lowest phonon bases.

Much attention has been devoted to the diagonal and off-diagonal coupling in organic materials^18^,^19^,^41^, but few of the existing work discuss the effect of their coexistence and competition. Empirical theories connect them respectively to the localization and delocalization, and then elucidate relevant experiments from individual perspective. In particular, as sketched in Fig. 1a, in the exciton state the electron is residing in a molecule which is recognized to be localized, while the CS state refers to a delocalization of the electron extending to a certain number of molecules. Following this language, we assign the well-established localized phase of TBSBM with the spin polarized along *z* direction to the exciton state, and the delocalized phase with spin polarized along *x* direction to the CS state. That is, the two levels of the spin (the spin up and down along *z* direction) mimic the local states in two molecules, respectively. At the critical point of these two phases, a critical phase has been claimed to exist^43^. In this phase, the spin is polarized along neither *z* nor *x* direction. To this end, we develop in this work the density matrix renormalization group (DMRG) algorithm by replacing the traditional asymmetrically optimized phonon basis (AOPB) with symmetrically optimized phonon basis (SOPB), as sketched in Fig. 1b. By this newly SOPB-adapted approach, we are able to explicitly determine the critical phase and thus the complete phase diagram in a numerical manner.

### Phase transition

**Figure 2:**
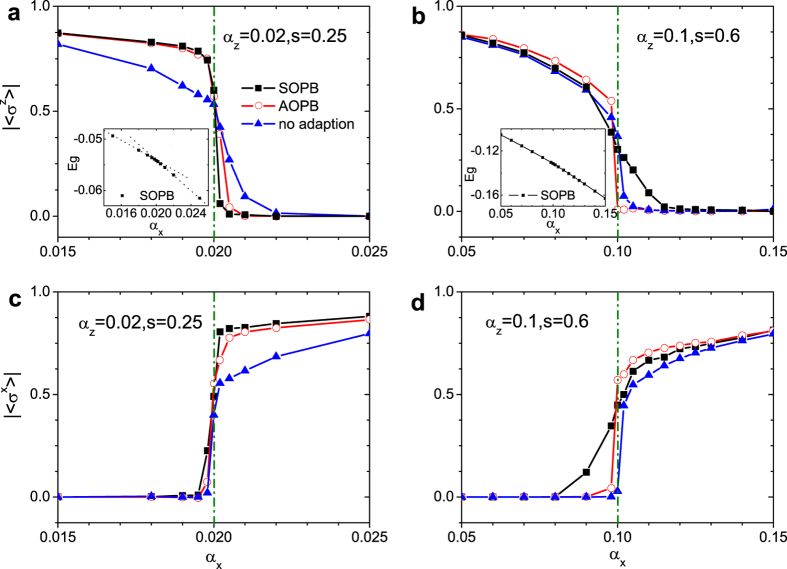
Spin coherence in two directions. 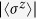
 and 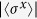 versus *α*_*x*_ for three cases of the basis with *s* = 0.25, *α*_*z*_ = 0.02 in (**a**, **c**) and *s* = 0.6, *α*_*z*_ = 0.1 in (**b**, **d**). The green dash-dot lines indicate the phase boundary at *α*_*x*_ = *α*_*z*_. The insets of (**a**,**b**) show the ground-state energy *E*_*g*_ versus *α*_*x*_. The dotted lines in the inset of (**a**) are guides for eyes of the linear relationship.

For the deep sub-Ohmic regime with *s* < 0.5, a transition from localized to delocalized phases has been discussed in our previous work^44^. Figure 2a shows 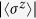 and the ground-state energy *E*_*g*_ for various values of *α*_*x*_ with *s* = 0.25 and *α*_*z*_ = 0.02. We compare three cases, with SOPB, with AOPB, and without any OPB adaption, and present results in the vicinity of the critical point. With 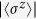 plotted as a function of the *α*_*x*_ for the three cases and compared to our previous work^44^,^45^,^46^, a much sharper decrease in 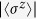 from a finite value to zero is found after the adaption of SOPB. An *α*_*x*_ increment of 0.0002 is taken around the critical point, which is numerically equivalent to being infinitesimal. In Fig. 2c, the 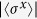 versus *α*_*x*_ is also displayed, and the relationship between them is almost inverse to that of 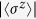 and *α*_*x*_. The critical point of *α*_*z*_ = *α*_*x*_ is essentially important as will be discussed shortly. In addition, the ground-state energy is shown in the inset for the case with SOPB with the parity symmetry fully considered. It is observed that there is an obvious kink at the critical point implying the phase transition. As the approach with SOPB gives rise to more precise results, it is implied that the phase transition here is of first order.

The shallow sub-Ohmic regime with 0.5 ≤ *s* < 1 presents even richer physics. It has been claimed that when *s* > 0.75, there is a so-called critical phase at *α*_*z*_ = *α*_*x*_^43^. From a mean-field analysis^45^,^46^, however, a similar phenomenon is found for *s* > 0.5 instead of *s* > 0.75. The discrepancy may be attributed to the fact that the mean-field theory is valid in the weak-coupling limit, while previous DMRG calculations are applicable in the relatively strong coupling regime. To resolve the problem, it is necessary to work with a greater parameter space. To this end, we first apply the SOPB-adapted approach to the case of *s* = 0.6. In Fig. 2b,d, we display 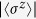 and 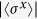 as a function of *α*_*x*_ obtained with SOPB, with AOPB and without OPB. It is found for the cases with AOPB and without OPB, both 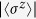 and 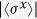 show a rather sudden change across the critical point. While adopting the SOPB method, the drops of 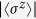 and 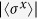 are less steep in the vicinity of the recognized critical point as compared to those from the other two cases. This effect implies that the transition is of higher order than that with the deep sub-Ohmic bath. Moreover, the curve of the ground-state energy shown in the inset of Fig. 2b is also smooth close to the critical point.

The smooth transition in the shallow sub-Ohmic regime points to the presence of a new phase. We follow the literature calling it the critical phase^43^. In this phase, both 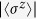 and 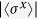 have finite values. The physical meaning is significant as both the local and nonlocal coherence (the diagonal and off-diagonal elements of the spin’s density matrix) which are so-called “stationary coherence”^47^ survive the coexistence of diagonal and off-diagonal vibronic coupling. This robust coherence highlights the essential role played by vibrational modes, that is, mutual cancelation of the two types of coupling to ensure the system to be free of decoherence. This vibrational coherence has been observed for the CT state^8^, implying the intrinsic connection between the critical phase and the CT process. To probe deeper into the vibrational coherence, we will carefully examine the bath modes.

### Parity symmetry

The symmetry in the Hamiltonian (1) is of paramount importance to the physical pictures. To facilitate discussion, we introduce the operators


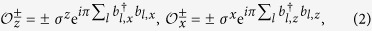


which commute with the Hamiltonian. Also of interest is their product, i.e.,


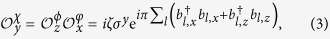


with *χ*, *ϕ, φ* = ± following the common rule of products. Together with the identity operator 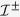, it can then be verified straightforwardly that the eight operators 
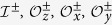
 form a non-abelian group *G*, and its center is represented by 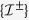. The factor group 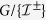 is an abelian group whose irreducible representations are given by four one-dimensional ones, indicating the U(1) symmetry when *α*_*z*_ = *α*_*x*_^42^. On the other hand, the two-dimensional representation of the non-abelian group *G*, characterized by a nontrivial central extension of its factor group, participates in the decomposition in irreducible representations, resulting in the 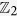 symmetry if *α*_*z*_ ≠ *α*_*x*_^45^,^46^. Subsequently, eigenstates of the system and, the ground state in particular, are doubly degenerate, a novel feature that allows specifications of the numerical precision in dealing with the symmetry.

### Order parameter and phase diagram

In practice, merely considering the spin coherence is insufficient to determine features of the CT state. Derivation of a more explicit quantity with the complete information of both the spin and the baths is required for this purpose. The argument from the group theory shows that the operators defined in Eq. (2) are the generators of the parity symmetry, with eigen-values of +1 or −1. Following the group-theoretical argument, hereafter we calculate two quantities, 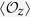 and 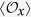, involving predictions of both the spin and the vibrational components. The calculation becomes possible because, as tested, the action of the operator 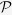 is precise based on the SOPB method.

For further clarity, we define an order parameter as 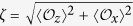. Since the spin is polarized along *z* (*x*) direction, in the localized (delocalized) phase with *α*_*z*_ ≠ *α*_*x*_, either 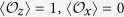 or 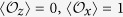, such that the quantity *ζ* will always be unity. Moreover, due to the spin polarization, the vibrational modes will be polarized along the respective direction as well, resulting in coherence loss in the dynamics, and consequently, inducing charge separation. In the critical phase, however, the significant phenomenon as we expected is that the coherence can not be quenched by the vibrational fluctuations, namely the phase is decoherence-free. We thus intuitively require the displacements of both diagonal and off-diagonal vibrational modes to simultaneously vanish. Let *X* and *Z* be the displacements of the vibrational mode in the two baths, respectively, which form a *X*-*Z* plane for all the modes. The continuous U(1) symmetry dominates the critical point with *α*_*z*_ = *α*_*x*_, implying that all the modes should be located in the circle around the origin of the *X*-*Z* plane. If and only if the displacements in both baths vanish, *ζ* emerges to be the unity, *ζ* will be smaller than one. The order parameter *ζ* then accounts for the condition of existence for the coherent and longstanding CT state.

**Figure 3:**
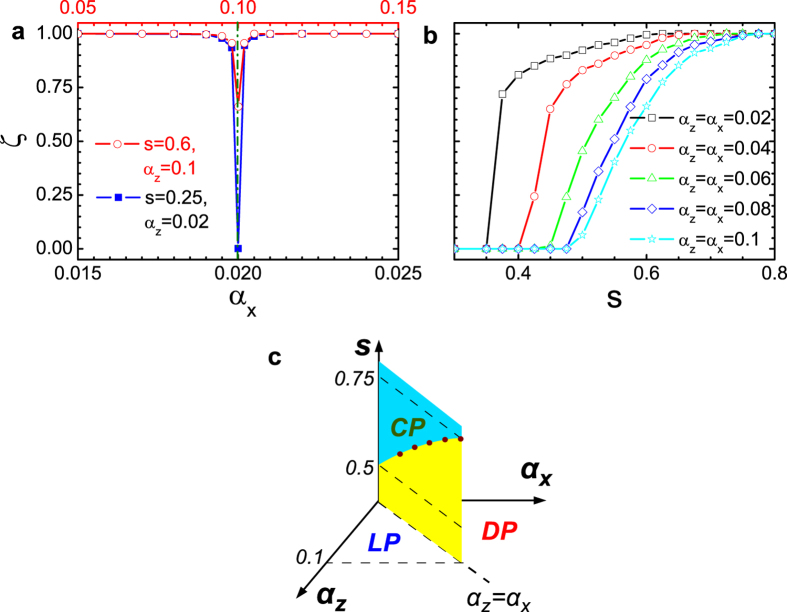
Order parameter *ζ* and phase diagram. (**a**) The order parameter *ζ* versus *α*_*x*_ changing from 0.015 to 0.035 for *s* = 0.25, *α*_*z*_ = 0.02 and ranging from 0.05 to 0.15 for *s* = 0.6, *α*_*z*_ = 0.1. The dash-dot line denotes the phase boundary. (**b**) *ζ* as a function of *s* for five sets of *α*_*z*_ and *α*_*x*_. (**c**) shows the phase diagram consisting of the localized phase (LP), the delocalized phase (DP) and the critical phase (CP).

Figure 3a shows the *α*_*x*_ dependence of *ζ* with *α*_*z*_ = 0.02 and 0.1 for *s* = 0.25 and 0.6, respectively. It is clear that for the deep sub-Ohmic regime, *ζ* undergoes a sudden change between 1 and 0 close to the critical point (*α*_*x*_ = *α*_*z*_). From the above arguments, this finding proves the absence of a critical phase and clarifies the existence of the first-order phase transition in the presence of the sub-Ohmic bath. More interesting is the shallow sub-Ohmic case, in which *ζ* is between 0 and 1 at the critical point implying the possible appearance of the critical phase. In order to see the point of appearance of the critical phase, we show in Fig. 3b the *s*-dependent *ζ* for five values of *α*_*z*_ right at *α*_*x*_ = *α*_*z*_. It is found that *ζ* vanishes if *s* is small, while it becomes unity if *s* is larger than a certain value. More importantly, the larger the *α*_*z*_, the larger the value of *s* for which *ζ* equals to one. In particular, for *α*_*z*_ = 0.1, the transition point where *ζ* becomes unity is located at *s* = 0.75 which is in agreement with that of the previous work^43^. On the other hand, a scaling analysis of *α*_*z*_ to the weak-coupling limit shows the transition point of *ζ* = 1 moving to *s* = 0.5 consistent with the expectation of the mean-field analysis^45^. Based on the transition points of *ζ*, we draw a phase diagram shown in Fig. 3c, where the boundaries of the localized, delocalized and critical phases are explicitly determined.

**Figure 4:**
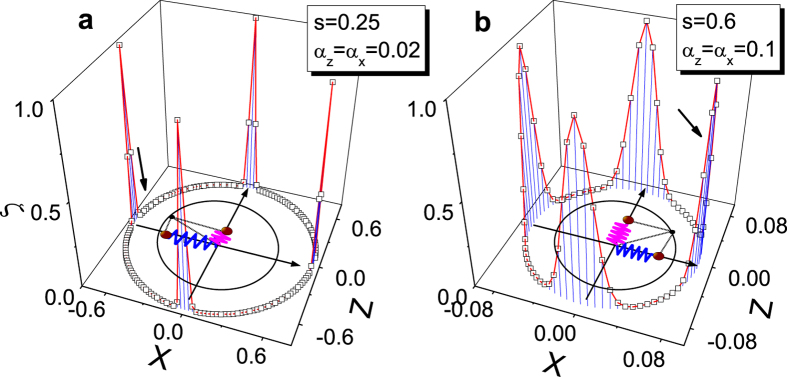
Order parameter *ζ* in the *X*-*Z* plane. The order parameter *ζ* calculated by the variational approach with rotational optimization shown in the *X*-*Z* plane for (**a**) *s* = 0.25, *α*_*z*_ = *α*_*x*_ = 0.02 and (**b**) *s* = 0.6, *α*_*z*_ = *α*_*x*_ = 0.1. The radius of the circle in the *X*-*Z* plane is determined by averaged displacements. The schematics in the *X*-*Z* plane shows the displacements with respect to the calculated ground states, which are pointed out by the arrows as well.

To ensure that the results are not influenced by numerical instability, we have also carried out the calculations based on variational theory^45^,^46^. Via the variational principle, we first produce a state with the lowest energy, which is not necessarily the ground state especially at the critical point with high numerical noise. Afterward, we rotate the boson states with a phase angle Θ following the same procedure as adopted for the DMRG calculations. This approach is justified because at the critical point the system obeys U(1) symmetry. By comparing the energies of the states, a real ground state can be found subsequently. Details of this approach are provided in the Appendix. For each state with Θ, we calculate the average displacements *X* and *Z* of both baths and the value of *ζ* accordingly, as shown in Fig. 4. We find that, the *X* and *Z* are largely reduced when *s* becomes large. This fulfills our expectation of the vanishing displacements of vibrational modes for the CT state. As well, *ζ* is found to vanish by the variational approach, lending support to our DMRG results. More importantly, in the deep sub-Ohmic regime, *ζ* completely vanishes for a majority of the cases, implying the states to be almost orthogonal to each other. The location of the real ground state has been indicated by the arrows in Fig. 4, in agreement with the DMRG results.

In the critical phase, the average displacements in the two baths vanish with the order parameter *ζ* equal to unity. Bearing in mind the finite values of the spin magnetization, there are two degenerate states with positive and negative spin averages entangled with relevant bath states of negative and positive displacements. All the bosonic modes together form the entangled state with the spin, which can be called “many-body entangled state”^48^. As studied in the previous work, the many-body entanglement is robust in the spin-boson model^49^, rendering the system decoherence-free. Therefore, the unity order parameter leads to the decoherence-free character of the critical phase via mutual cancellation of diagonal and off-diagonal coupling.

## Discussion

We have studied critical phase of the TBSBM in both the deep and shallow sub-Ohmic regimes. Both the spin coherence and the ground-state energy for *s* = 0.25 change significantly, supporting a first-order phase transition, while for *s* = 0.6 the changes become smoother indicating a higher order phase transition. By defining a new order parameter *ζ*, various features of the critical phase are revealed and the phase diagram is explicitly obtained. It is also found that through the SOPB adaptation, the numerical precision is greatly improved in both sub-Ohmic regimes. Clearly the SOPB-adapted DMRG algorithm is well-suited to handle the complexity of the phase transitions in the SBM. This robust approach is expected to be extended to tackle other issues, such as the real-time dynamics of the SBM.

To make connections to the charge separation in PSCs, four remarks are in order. The first is on the existence and robustness of the critical phase when the diagonal and off-diagonal coupling are in fine balance. The critical phase falls between the localized and the delocalized phase, similar to the CT state. We thus conclude that the appearance of the CT state hinges on the competition between intra- and inter-molecular vibrational modes. More importantly, the decoherence-free character originated from the mutual cancellation of the intra- and inter-molecular coupling leads to the lasting coherence in the CT state. Secondly, as the vibrational modes are coupled to the photo-excitations, it is expected that frequent conversion among the exciton, the CT state, and the CS state occurs naturally. In particular, when an infrared optical excitation is injected and coupled to the intermolecular vibrational modes, there is a good chance for the delocalized phase to emerge leading to charge separation. Such a picture may help interpret the results of the pump-push-probe experiment^3^. Thirdly, as the three phases are not affected by the spin energy, it is inferred that the charge separation process may be insensitive to detailed molecular structures, which may also help explain why the internal quantum efficiency does not depend on the level alignment^4^. Finally, one of the presumptions of the present work is that the ground state structure of the model dominates the dynamical behavior. Although the ground state is the main concern in this work, it is reasonable to extend our conclusions to the case of room temperature, since the characteristic energy scale of excitons in organic materials is of the order ~100 meV which is much larger than the thermal energy at room temperature^16^.

## Methods

### Density matrix renormalization group

The DMRG algorithm is a powerful numerical technique to study the low-lying states in strongly coupled, one-dimensional systems^50^. Similar to that for the numerical renormalization group^51^ method, the orthogonal polynomials theory can be employed to map the SBM to a one-dimensional chain with only nearest-neighbor coupling^52^, allowing one to straightforwardly adapt the DMRG method to study the SBM with only model-free approximations. Over the last few years, this approach has been extensively used for detailed studies of the phase transition of SBM^42^,^43^,^44^,^45^,^53^,^54^. Our recent work was devoted to the TBSBM to investigate the phase transition in a comprehensive manner^44^,^45^,^46^,^55^. We have also examined the dynamics of SBM with the time-dependent DMRG (t-DMRG) algorithm, compared it with two other established methods, and demonstrated that a unitary transformation for the state yields reliable, accurate results^55^,^56^.

We thus proceed to develop a DMRG algorithm to deal with the model of high symmetry. Widely used to study the SBM and related models^42^,^43^,^44^,^45^,^46^,^53^,^54^,^56^, the DMRG approach starts with the discretization of the boson modes, and employs the orthogonal polynomials theory to represent the renormalized modes by a set of boson sites^52^, with a transformed Hamiltonian


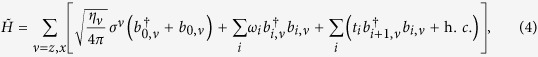


where *η*_*ν*_ is the renormalized coupling calculated from 
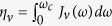
. Herein, *ω*_*i*_ and *t*_*i*_ are the frequency and the hopping integral for the *i*-th site of bosons, respectively, and the expressions for them can be found in refs. 44,45,56. In all the calculations we have carried out, the number of transformed bosonic site is set to be 50, the number of bare phonon basis is 16, and the DMRG truncating number is 64. Within these parameters, the error is reduced to below 10^−5^. The computation is time-consuming, e.g., on a single 2.13 GHz processor one run for a set of model parameters needs more than one hundred hours of CPU time.

### Optimal phonon basis

Despite discretization of the spectral densities, the number of the bare phonon basis in the local Fock space for each renormalized boson mode is still infinite, hindering numerical calculations. A remedy is to truncate the Fock space and retain a finite number of bare phonon states for each mode, resulting in a so-called restricted phonon basis. Previous tests^56^ reveal that the restricted basis method is applicable for the SBM away from the critical point, but fails to capture the phase-transition properties in the vicinity of the critical point. An approach employing an optimal phonon basis (OPB) was adopted by Zhang *et al.*, yielding improved results^57^. The calculation procedure of the OPB-adapted DMRG algorithm can be briefly summed up as follows. Firstly, we apply the usual DMRG procedure with restricted basis to obtain the state with the lowest energy which may be a rough estimate of the true ground state energy of the system^50^. To accelerate the computations, it is useful to add a very small bias *ε* to the system in this step to lift the degeneracy. The sign of the bias is not important in this step, because in the steps later on both degenerate states will be obtained and the influence of the small bias will be eliminated by the scaling analysis. Secondly, the DMRG iteration is continued, and the basis of the Fock space on the left single site is replaced by the bare phonon one during each step of the iteration. The number of bare basis (*N*_*B*_) is much larger than that of the restricted basis (*N*_*R*_), ensuring the convergency of the local energy. Thirdly, a new state with a much lowered energy with respect to the bare basis is obtained. Based upon this state, we calculate the reduced density matrix of the left single site and follow the idea of DMRG to discard those bases with low probability. The new reduced basis for the local Fock space is the OPB, carrying as much information as the bare phonon basis. After one iteration, all the boson sites are optimized with OPB, and then the system energy is minimized using the usual DMRG procedure. Finally, a state with an energy very close to that of the real ground state is obtained, with which we can calculate all desired observables.

### Symmetrically optimized procedure

Although the OPB approach is highly efficient and has been utilized for a variety of models^58^,^59^,^60^,^61^, a serious numerical problem arises in the context of the TBSBM wherein the symmetry is broken in the numerical procedure hindering the analysis of the phase transition^42^. A seemingly simple solution is to add an infinitesimal bias and to approach the critical point asymptotically. However, this would lead to a disastrously large difference in symmetry between *α*_*z*_ ≠ *α*_*x*_ and *α*_*z*_ = *α*_*x*_ (the critical point) as discussed earlier. To circumvent this dilemma, we propose two techniques to enforce the model symmetry. It is realized that in the implementation of the OPB approach, many low probability states are discarded. As sketched in Fig. 1b, e.g., if the sign of the calculated boson displacement is negative, those states with a positive sign must be eliminated as they are almost orthogonal to the negative-displacement states. To recover the symmetry, therefore, the information in the eliminated states must be fed into the reduced density matrix. In particular, if 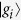 is the calculated ground state with *i* as the index of the left free site, we apply the parity operator 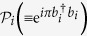 onto 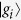, obtaining 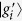 with an opposite sign of the boson displacement on *i*-th site. This is numerically feasible as the *i*-th site is the one that has the Fock space based upon the bare phonon basis. With the two states obtained, the reduced density matrix of the *i*-th site is calculated by


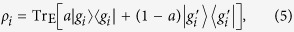


where Tr_E_ denotes the partial trace over all the sites except the *i*-th one and *a* is the portion of the state 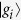 in the reduced density matrix. In the absence of bias, it is intuitive to set *a* to 0.5. We then name the new basis obtained from the adapted reduced density matrix as the “symmetrically optimized phonon basis (SOPB),” and accordingly, the conventional OPB is called the “asymmetrically optimized phonon basis (AOPB).” We will show that the optimization procedure based on the SOPB yields more accurate results.

With one run of the DMRG algorithm, one degenerate ground state is obtained, and with a second run, we may obtain another, allowing for a linear combination of the two^42^. However, symmetry breaking still persists reducing the accuracy and the efficiency. A sophisticated approach would then be to apply the operators (2) to the calculated ground state. In order to implement this operation, through the parity operator 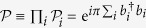, a unitary transformation on all bosonic modes must be applied to flip the sign of displacements. As the bases of the boson modes have been symmetrically optimized, however, the number operator 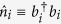 ceases to be diagonal. Moreover, the two degenerate states are almost orthogonal to each other, so that a simple diagonalization for 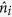 can no longer guarantee numerical precision. In this context, we introduce a more numerically friendly treatment of the parity operator by recasting it in the form


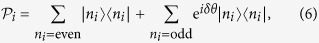


where 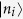 is the eigenstate of the number operator 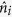 at *i*-th site, and *δθ* is a small angle. Herein, following the t-DMRG algorithm^62^, the angle *π* is divided into many small steps (*δθ*) and the operator is applied incrementally to the ground state. The operator does not act on the even-number states, while for the odd-number states one can make 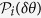 act cumulatively onto the state until a certain angle *θ*. If *θ* = *π* the action is equivalent to that of the parity operator, and if *θ* = 2*π* it is an identity operator. With this approach, reliable results can be obtained for all model parameters.

### Examination of the precision

In order to examine the reliability of our numerical approach, the following test can be performed. The operator 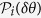 can be applied step by step onto the ground state 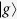 to obtain a new state 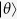. As discussed, the states with opposite signs displacements are orthogonal to each other. For example, if *θ* = *π*, the new state is orthogonal to the ground state, i.e., 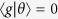; if *θ* = 2*π*, the two states are identical. Therefore, if the information of the reversed-sign state is not taken into account in the numerical optimization, the action of the operator 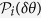 will give rise to results completely out of expectation. The examination of this operator is a critical criterion to determine whether our numerical approach is able to attain the double degeneracy and thus the precise location of the phase transition.

**Figure 5:**
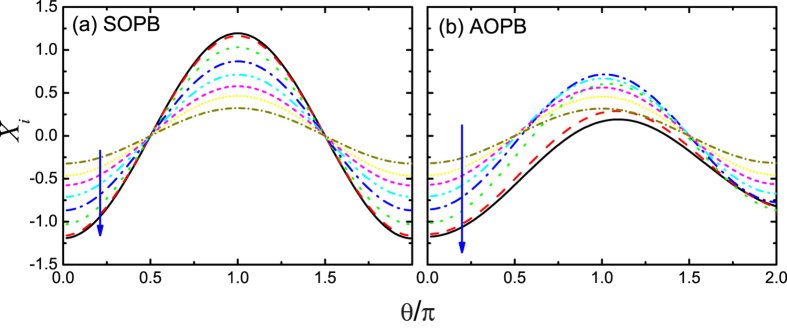
The effective displacement of boson on the odd sites that are closest to the spin versus the rotation angle with (a) the SOPB and (b) the AOPB. The parameters are *s* = 0.25, *α*_*z*_ = *α*_*x*_ = 0.02, *N*_*B*_ = 16. The arrow indicates the orientation away from the spin.

Based upon the state 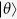, we calculate the displacement of each boson mode 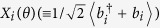 with both SOPB and AOPB, and results are shown in Fig. 5 for *α*_*x*_ = 0.02 and 8 odd sites that are closest to the spin. It is found that for the case with SOPB, if *θ* = *π*, signs of the displacements are all flipped, while, if *θ* = 2*π*, the signs all remain unchanged. On the other hand, however, for the case with AOPB, the displacements are all smaller than those of SOPB. More importantly, after the rotation of 2*π* angle the displacements do not return to the original points, implying the AOPB curves are incorrect when *θ* becomes large. We also calculate the energy (not shown), which is directly related to the displacement of the boson modes, and similar behavior is observed for the two cases. These results demonstrate the great improvements our numerical approach achieved by explicitly taking into account the parity symmetry. In addition, the difference in the displacement between *θ* = 0 and *θ* = 2*π* is a perfect measure of the numerical precision, and here the difference is found to be smaller than 10^−5^. We have also checked the value of 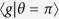 which is of the same order of magnitude.

## Additional Information

**How to cite this article**: Yao, Y. *et al.* Competition between diagonal and off-diagonal coupling gives rise to charge-transfer states in polymeric solar cells. *Sci. Rep.* **5**, 14555; doi: 10.1038/srep14555 (2015).
